# Assessment of risk factors associated with common chronic diseases in an adult population

**DOI:** 10.6026/973206300220839

**Published:** 2026-02-28

**Authors:** Nishant Kakkad, Shuchi Shah, Naresh Parmar, Jitendra Patel, Kishan Dineshbhai Rabadiya

**Affiliations:** 1 Department of Biochemistry, Ananya College of Medicine and Research, Kalol, Gujarat, India; 2 Department of Anesthesiology, Dr N D Desai Faculty of Medical Science and Research, Dharmsinh Desai University, Nadiad, Gujarat, India; 3 Department of Physiology, All India Institute of Medical Sciences, Rajkot, Gujarat, India; 4 Department of Physiology, GMERS Medical College, Vadnagar, Gujarat, India; 5 Department of Physiology, Swaminarayan Institute of Medical Sciences & Research, Kalol, Gujarat, India

**Keywords:** Chronic diseases, risk factors, cardiovascular disease, diabetes mellitus, lifestyle modification, prevention

## Abstract

Chronic diseases remain the leading contributors to global morbidity and mortality, largely driven by modifiable risk factors. Therefore, it is of interest to evaluate the prevalence and clustering of major chronic disease risk factors among 1,248 adults aged 25-74 years attending primary healthcare centers between March 2022 and August 2024. Demographic, behavioral, anthropometric and biochemical parameters were assessed across cardiovascular disease, type 2 diabetes mellitus, chronic respiratory disease and chronic kidney disease. Physical inactivity (68.4%), overweight/obesity (61.2%) and dyslipidemia (54.8%) were the most prevalent risk factors, with significant clustering among individuals with established chronic conditions. Obesity, hypertension, smoking and physical inactivity independently predicted chronic disease presence, underscoring the need for integrated lifestyle-focused prevention and early intervention strategies.

## Background:

Chronic non-communicable diseases have emerged as the predominant global health challenge of the twenty-first century, accounting for approximately 71% of all deaths worldwide and imposing substantial burdens on healthcare systems, economies and individual quality of life [1]. The epidemiological transition from communicable to non-communicable disease predominance has accelerated across developed and developing nations alike, driven by demographic shifts, urbanization and lifestyle changes accompanying modernization [2]. Cardiovascular diseases, diabetes mellitus, chronic respiratory diseases and malignancies collectively constitute the major chronic disease categories, sharing common modifiable risk factors that present opportunities for primary prevention [3]. The World Health Organization has emphasized that a significant proportion of chronic disease burden is attributable to modifiable behavioral and metabolic risk factors, including tobacco use, harmful alcohol consumption, physical inactivity, unhealthy diet and resultant overweight and obesity [4]. The pathophysiological mechanisms linking behavioral risk factors to chronic disease development involve complex interactions between metabolic dysregulation, chronic inflammation, oxidative stress and endothelial dysfunction [5]. Hypertension, dyslipidemia and hyperglycemia represent intermediate metabolic risk factors that mediate the relationship between lifestyle behaviors and end-organ disease manifestation [6]. Population-based studies have consistently demonstrated dose-response relationships between risk factor exposure and chronic disease incidence, with cumulative risk burden substantially exceeding additive predictions [7]. The clustering of multiple risk factors within individuals amplifies overall disease probability through synergistic biological mechanisms that accelerate pathological processes [8]. Socioeconomic determinants profoundly influence risk factor distribution, with disadvantaged populations experiencing disproportionate exposure to obesogenic environments, tobacco marketing and barriers to healthy lifestyle adoption [9]. Educational attainment, income level and occupational characteristics correlate significantly with chronic disease risk profiles across diverse populations [10]. Contemporary research has increasingly recognized the importance of life-course perspectives in chronic disease epidemiology, acknowledging that early-life exposures and developmental programming influence adult disease susceptibility [11]. Nevertheless, adult risk factor modification remains effective for disease prevention and management, supporting continued investment in population-level and individual-level intervention strategies [12]. Regional variations in chronic disease burden and risk factor prevalence necessitate context-specific assessment to inform locally appropriate prevention priorities [13]. Standardized surveillance methodologies enable cross-population comparisons while identifying unique risk profiles requiring targeted intervention approaches [14]. Despite extensive global research on chronic disease risk factors, comprehensive local assessments integrating behavioral, anthropometric and biochemical parameters remain insufficiently implemented in many healthcare settings [15]. Such assessments provide essential evidence for healthcare planning, resource allocation and intervention program development. Therefore, it is of interest to comprehensively assess the prevalence and clustering patterns of major risk factors associated with common chronic diseases among adults attending primary healthcare facilities. Secondary objectives included examination of sociodemographic correlates of risk factor burden and identification of high-risk subpopulations requiring prioritized intervention.

## Materials and Methods:

## Study design and setting:

This community-based cross-sectional study was conducted across six primary healthcare centers serving diverse urban and semi-urban populations between March 2022 and August 2024. The participating centers were selected through stratified random sampling to ensure socioeconomic representativeness of the catchment population.

## Study population and sampling:

The target population comprised adults aged 25-74 years residing within designated catchment areas. Systematic random sampling was employed, with every third eligible patient attending participating centers during the study period invited to participate. Sample size was calculated based on anticipated chronic disease prevalence of 25%, precision margin of 3% and 95% confidence level, yielding a minimum requirement of 1,200 participants.

## Inclusion criteria:

[1] Age between 25 and 74 years

[2] Permanent residence within catchment area for at least 12 months

[3] Ability to provide informed consent

[4] Willingness to complete all study assessments

## Exclusion criteria:

[1] Pregnancy or breastfeeding

[2] Acute illness requiring immediate medical attention

[3] Severe cognitive impairment precluding reliable response

[4] Terminal illness with life expectancy less than 6 months

[5] Previous participation in the study

## Data collection instruments:

## Structured questionnaire:

A standardized questionnaire adapted from the WHO STEPwise approach to surveillance was administered through face-to-face interviews by trained research assistants. The questionnaire encompassed:

[1] Demographic variables: Age, sex, marital status, educational attainment, occupation, household income and residence type.

[2] Behavioral risk factors: Current and former tobacco use, frequency and quantity of alcohol consumption, dietary patterns including fruit, vegetable, salt and fat intake and physical activity levels assessed using the Global Physical Activity Questionnaire.

[3] Medical history: Physician-diagnosed chronic conditions, current medications, family history of chronic diseases and healthcare utilization patterns.

## Physical measurements:

Anthropometric and physiological measurements were obtained following standardized protocols:

[1] Height: Measured using calibrated stadiometer to nearest 0.1 cm, without shoes.

[2] Weight: Measured using digital scale to nearest 0.1 kg, in light clothing.

[3] Waist circumference: Measured at midpoint between lowest rib and iliac crest using non-elastic tape.

[4] Blood pressure: Measured using validated automated sphygmomanometer after 5-minute seated rest, with average of two readings recorded.

## Biochemical assessments:

Fasting venous blood samples were collected for laboratory analysis including:

[1] Fasting plasma glucose

[2] Glycated hemoglobin (HbA1c)

[3] Total cholesterol, HDL-cholesterol, LDL-cholesterol, triglycerides

[4] Serum creatinine with estimated glomerular filtration rate

## Risk factor definitions:

[1] Tobacco use: Current smokers defined as individuals smoking any tobacco product within past 30 days.

[2] Harmful alcohol use: Consumption exceeding 14 standard drinks per week for males or 7 for females.

[3] Physical inactivity: Less than 150 minutes of moderate-intensity activity or 75 minutes of vigorous activity per week.

[4] Overweight/Obesity: Body mass index ≥25 kg/m^2^ (overweight) or ≥30 kg/m^2^ (obesity).

[5] Central obesity: Waist circumference >102 cm (males) or >88 cm (females).

[6] Hypertension: Systolic blood pressure ≥140 mmHg, diastolic blood pressure ≥90 mmHg, or current antihypertensive medication use.

[7] Dyslipidemia: Total cholesterol ≥200 mg/dL, LDL-cholesterol ≥130 mg/dL, HDL-cholesterol <40 mg/dL (males) or <50 mg/dL (females), triglycerides ≥150 mg/dL, or current lipid-lowering medication.

[8] Dysglycemia: Fasting glucose ≥100 mg/dL, HbA1c ≥5.7%, or diagnosed diabetes mellitus.

## Chronic disease definitions:

Chronic diseases were identified through self-reported physician diagnosis, medication use, or study measurements meeting diagnostic criteria:

[1] Cardiovascular disease: History of myocardial infarction, stroke, angina, or heart failure.

[2] Type 2 diabetes: Fasting glucose ≥126 mg/dL, HbA1c ≥6.5%, or current antidiabetic medication.

[3] Chronic kidney disease: Estimated GFR <60 mL/min/1.73m^2^.

[4] Chronic respiratory disease: Physician-diagnosed asthma or chronic obstructive pulmonary disease with current symptoms or treatment.

## Statistical analysis:

Data were entered using double-entry verification and analyzed using SPSS version 27.0 and R version 4.2.0. Descriptive statistics included means with standard deviations for continuous variables and frequencies with percentages for categorical variables. Distribution normality was assessed using Kolmogorov-Smirnov test. Group comparisons employed independent samples t-test or Mann-Whitney U test for continuous variables and chi-square test or Fisher's exact test for categorical variables. Prevalence estimates were calculated with 95% confidence intervals. Risk factor clustering was examined through frequency distribution of number of concurrent risk factors and chi- square analysis of clustering patterns. Logistic regression models identified independent predictors of chronic disease presence, with odds ratios and 95% confidence intervals reported. Model fit was assessed using Hosmer-Lemeshow test. Statistical significance was defined as p < 0.05.

## Results:

A total of 1,248 adults completed all study assessments, representing a participation rate of 87.3% among those approached. The study population comprised 542 males (43.4%) and 706 females (56.6%), with mean age of 48.7 ± 13.4 years. Educational attainment varied considerably, with 18.2% having completed higher education and 23.6% having primary education or less. Chronic disease prevalence was substantial: type 2 diabetes (16.8%), hypertension as a condition (38.4%), cardiovascular disease (8.6%), chronic kidney disease (7.2%) and chronic respiratory disease (9.4%). Overall, 412 participants (33.0%) had at least one diagnosed chronic condition (Table 1). Behavioral and metabolic risk factors demonstrated high prevalence across the study population. Physical inactivity was the most common behavioral risk factor (68.4%), followed by insufficient fruit and vegetable consumption (62.8%). Current tobacco use was reported by 24.6% of participants, with higher rates among males (34.8%) compared to females (16.8%, p < 0.001). Overweight and obesity affected 61.2% of participants (38.4% overweight, 22.8% obese), with central obesity present in 48.6%. Hypertension was detected in 38.4%, while dyslipidemia affected 54.8% of participants. Dysglycemia (prediabetes or diabetes) was identified in 32.4% (Table 2). Analysis of concurrent risk factors revealed substantial clustering within individuals. The mean number of risk factors was 3.8 ± 1.9 in the overall population, significantly higher among those with chronic disease (4.9 ± 1.7) compared to those without (3.2 ± 1.8, p < 0.001). Only 4.8% of participants had no identifiable risk factors, while 28.6% had five or more concurrent risk factors. Risk factor clustering demonstrated strong association with chronic disease presence. Among participants with 0-1 risk factors, chronic disease prevalence was 8.4%, increasing progressively to 62.8% among those with five or more risk factors (p for trend < 0.001) (Table 3). Multivariate logistic regression analysis adjusting for age, sex and socioeconomic variables identified independent predictors of chronic disease presence. Sex-stratified analysis revealed differential risk factor impacts. Among males, smoking demonstrated stronger association with chronic disease (OR: 2.84) compared to females (OR: 1.92). Conversely, obesity showed stronger association among females (OR: 3.68) than males (OR: 2.76). Age-stratified analysis indicated that modifiable risk factors exerted greater relative influence among younger adults (25-44 years), where lifestyle factors accounted for a larger proportion of chronic disease variance compared to older age groups where age-related factors predominated.

## Discussion:

This comprehensive assessment of chronic disease risk factors in an adult population reveals alarming prevalence of modifiable determinants and substantial clustering of multiple risk factors within individuals. The findings underscore the urgent need for integrated prevention strategies targeting lifestyle modification across the population while prioritizing high-risk subgroups for intensive intervention. The observed prevalence of physical inactivity exceeding two-thirds of the population reflects contemporary patterns of sedentary behavior associated with urbanization, occupational changes and technological advancement [16]. Insufficient physical activity has been established as an independent risk factor for cardiovascular disease, type 2 diabetes and certain malignancies, with global estimates suggesting it contributes to approximately 3.2 million deaths annually [17]. Overweight and obesity prevalence approaching two-thirds of participants aligns with worldwide trends of increasing adiposity, driven by energy-dense dietary patterns and reduced physical activity [18]. The metabolic consequences of excess adiposity, including insulin resistance, chronic inflammation and dyslipidemia, mediate substantial proportions of chronic disease risk attributed to obesity [19]. The strong independent association between obesity and chronic disease presence, with odds ratios exceeding 3.0, reinforces the central importance of weight management in disease prevention [20]. Central obesity demonstrated additional predictive value beyond general obesity measures, consistent with evidence that visceral adipose tissue accumulation carries particular metabolic and cardiovascular risk [21]. Tobacco use prevalence, while lower than some regional comparisons, remains a significant public health concern given the dose-response relationship between smoking and multiple chronic diseases [22]. The elevated chronic disease risk among current smokers observed in this study corresponds with established evidence of tobacco-related pathophysiology affecting cardiovascular, respiratory and metabolic systems [23].

Hypertension emerged as a potent predictor of chronic disease presence, reflecting its role as both a chronic condition and a risk factor for cardiovascular events, chronic kidney disease and cerebrovascular disease [24]. The substantial proportion of participants with elevated blood pressure highlights the importance of population-level blood pressure screening and management programs [25]. Dyslipidemia prevalence exceeding half the study population represents a major modifiable contributor to cardiovascular risk [26]. The relationship between lipid abnormalities and atherosclerotic disease progression is well-characterized, supporting aggressive lipid management for disease prevention [27]. The pronounced risk factor clustering observed, with nearly one-third of participants harboring five or more concurrent risk factors, illustrates the phenomenon of risk aggregation within individuals [28]. Shared behavioral determinants and biological pathways connecting risk factors produce clustering patterns that substantially amplify overall disease probability beyond additive effects [29]. Socioeconomic gradients in risk factor distribution were evident, with lower educational attainment and income associated with increased chronic disease burden [30]. These disparities reflect differential exposure to health-promoting environments and resources, limited health literacy and structural barriers to healthy lifestyle adoption [31]. The family history associations identified in this study support genetic contributions to chronic disease susceptibility while also reflecting shared environmental and behavioral patterns within families [32]. Integration of family history assessment into risk stratification enhances identification of individuals warranting enhanced surveillance and intervention [33]. The differential impact of specific risk factors across sex and age subgroups has implications for targeted intervention design [34]. Sex-specific prevention messaging and age-appropriate intervention modalities may enhance effectiveness of chronic disease prevention programs [35]. Study limitations include the cross-sectional design precluding causal inference, potential selection bias from healthcare facility-based recruitment, reliance on self-reported behavioral data susceptible to recall and social desirability biases and geographic specificity potentially limiting generalizability. Additionally, certain chronic conditions may have remained undiagnosed among participants classified as disease-free. The findings have direct implications for public health policy and clinical practice. Population-level interventions addressing obesogenic environments, tobacco availability and physical activity infrastructure should be complemented by individual-level counseling integrated into primary healthcare delivery [36]. Risk stratification algorithms incorporating multiple factors can guide resource allocation toward high-risk individuals requiring intensive intervention [37].

## Conclusion:

This study demonstrates a high prevalence of modifiable chronic disease risk factors among adults, with physical inactivity, overweight/obesity and dyslipidemia being most common and frequent clustering markedly increasing disease likelihood. Obesity, hypertension, smoking and physical inactivity emerged as the strongest independent predictors of chronic disease, underscoring the dominant role of lifestyle-related determinants. These findings support integrated, equity-focused prevention strategies within primary healthcare that emphasize systematic risk factor screening, targeted lifestyle interventions and early risk reduction to mitigate chronic disease burden.

## Figures and Tables

**Table 1:** Sociodemographic characteristics of study participants by chronic disease status

**Characteristic**	**Total (n=1,248)**	**Without Chronic Disease (n=836)**	**With Chronic Disease (n=412)**	**p-value**
Age (years), mean± SD	48.7± 13.4	44.2± 12.1	57.8± 11.6	<0.001
Age Groups, n (%)				<0.001
25-34 years	218 (17.5)	189 (22.6)	29 (7.0)	
35-44 years	286 (22.9)	224 (26.8)	62 (15.0)	
45-54 years	298 (23.9)	198 (23.7)	100 (24.3)	
55-64 years	276 (22.1)	148 (17.7)	128 (31.1)	
65-74 years	170 (13.6)	77 (9.2)	93 (22.6)	
Sex, n (%)				0.024
Male	542 (43.4)	346 (41.4)	196 (47.6)	
Female	706 (56.6)	490 (58.6)	216 (52.4)	
Education, n (%)				<0.001
Primary or less	294 (23.6)	168 (20.1)	126 (30.6)	
Secondary	726 (58.2)	498 (59.6)	228 (55.3)	
Higher education	228 (18.2)	170 (20.3)	58 (14.1)	
Marital Status, n (%)				0.008
Married/Cohabiting	864 (69.2)	562 (67.2)	302 (73.3)	
Single	224 (17.9)	168 (20.1)	56 (13.6)	
Divorced/Widowed	160 (12.8)	106 (12.7)	54 (13.1)	
Employment, n (%)				<0.001
Employed	684 (54.8)	512 (61.2)	172 (41.7)	
Unemployed	198 (15.9)	142 (17.0)	56 (13.6)	
Retired	248 (19.9)	108 (12.9)	140 (34.0)	
Homemaker	118 (9.5)	74 (8.9)	44 (10.7)	
Income Level, n (%)				0.003
Low	412 (33.0)	256 (30.6)	156 (37.9)	
Middle	598 (47.9)	412 (49.3)	186 (45.1)	
High	238 (19.1)	168 (20.1)	70 (17.0)	

**Table 2:** Prevalence of risk factors by chronic disease status

**Risk Factor**	**Total n (%)**	**Without Chronic Disease n (%)**	**With Chronic Disease n (%)**	**p-value**
**Behavioral Factors**				
Current smoking	307 (24.6)	178 (21.3)	129 (31.3)	<0.001
Former smoking	186 (14.9)	108 (12.9)	78 (18.9)	0.006
Harmful alcohol use	168 (13.5)	98 (11.7)	70 (17.0)	0.012
Physical inactivity	854 (68.4)	524 (62.7)	330 (80.1)	<0.001
Low fruit/vegetable intake	784 (62.8)	498 (59.6)	286 (69.4)	0.001
High salt intake	542 (43.4)	342 (40.9)	200 (48.5)	0.012
High saturated fat intake	468 (37.5)	298 (35.6)	170 (41.3)	0.056
**Anthropometric Factors**				
Overweight (BMI 25-29.9)	479 (38.4)	318 (38.0)	161 (39.1)	0.724
Obesity (BMI ≥30)	285 (22.8)	142 (17.0)	143 (34.7)	<0.001
Central obesity	607 (48.6)	342 (40.9)	265 (64.3)	<0.001
**Metabolic Factors**				
Hypertension	479 (38.4)	198 (23.7)	281 (68.2)	<0.001
Elevated total cholesterol	524 (42.0)	312 (37.3)	212 (51.5)	<0.001
Elevated LDL-cholesterol	486 (38.9)	286 (34.2)	200 (48.5)	<0.001
Low HDL-cholesterol	398 (31.9)	238 (28.5)	160 (38.8)	<0.001
Elevated triglycerides	412 (33.0)	234 (28.0)	178 (43.2)	<0.001
Any dyslipidemia	684 (54.8)	398 (47.6)	286 (69.4)	<0.001
Dysglycemia	404 (32.4)	168 (20.1)	236 (57.3)	<0.001
**Family History**				
CVD family history	386 (30.9)	228 (27.3)	158 (38.3)	<0.001
Diabetes family history	342 (27.4)	198 (23.7)	144 (35.0)	<0.001

**Table 3:** Multivariate analysis of risk factors associated with chronic disease

**Risk Factor**	**Adjusted Odds Ratio**	**95% Confidence Interval**	**p-value**
Age (per 10-year increase)	1.86	1.64 - 2.11	<0.001
Male sex	1.28	0.98 - 1.67	0.068
Low educational attainment	1.42	1.08 - 1.87	0.012
Current smoking	2.46	1.82 - 3.32	<0.001
Harmful alcohol use	1.68	1.18 - 2.39	0.004
Physical inactivity	2.18	1.62 - 2.94	<0.001
Low fruit/vegetable intake	1.34	1.02 - 1.76	0.036
Obesity (BMI ≥30)	3.24	2.38 - 4.41	<0.001
Central obesity	1.78	1.34 - 2.36	<0.001
Hypertension	2.89	2.18 - 3.83	<0.001
Dyslipidemia	1.92	1.46 - 2.52	<0.001
Dysglycemia	2.64	1.98 - 3.52	<0.001
Family history of CVD	1.48	1.12 - 1.95	0.006
Family history of diabetes	1.36	1.02 - 1.81	0.038
Number of risk factors (≥5 vs <3)	4.82	3.24 - 7.16	<0.001
Model demonstrated adequate fit
(Hosmer-Lemeshow p = 0.342) with area under
curve of 0.826 (95% CI: 0.802-0.850) for
chronic disease prediction.
